# Extract Enriched in Flavan-3-ols and Mainly Procyanidin Dimers Improves Metabolic Alterations in a Mouse Model of Obesity-Related Disorders Partially via Estrogen Receptor Alpha

**DOI:** 10.3389/fphar.2018.00406

**Published:** 2018-04-24

**Authors:** Daniela Leonetti, Raffaella Soleti, Nicolas Clere, Luisa Vergori, Caroline Jacques, Lucie Duluc, Catherine Dourguia, Maria C. Martínez, Ramaroson Andriantsitohaina

**Affiliations:** ^1^INSERM UMR1063, Stress Oxydant et Pathologies Métaboliques, Faculté de Santé, UNIV Angers, Université Bretagne Loire, Angers, France; ^2^Centre Hospitalier Universitaire d’Angers, Angers, France

**Keywords:** estrogen receptor α, metabolic disorders, obesity, grape seed extract, vascular disorders

## Abstract

Red wine polyphenol extracts improve cardiovascular and metabolic disorders linked to obesity. Their vascular protection is mediated by the activation of the alpha isoform of the estrogen receptor (ERα). In the present study, we explored the effects of a grape seed extract (GSE) enriched in the flavan-3-ols procyanidin dimers on obesity-related cardiovascular and metabolic disorders; with a particular interest in the role/contribution of ERα. Ovariectomized wild type or ERα knockout (KO) mice were fed with standard or western diet, supplemented or not with GSE, for 12 weeks. Their body weight was monitored throughout the study, and an echocardiography was performed at the end of the treatment. Blood and tissues were collected for biochemical and functional analysis, including nitric oxide and oxidative stress measurement. Vascular reactivity and liver mitochondrial complexes activity were also analyzed. In western diet-fed mice, GSE reduced adiposity, plasma triglycerides, and oxidative stress in the heart, liver, adipose and skeletal tissues; but did not improve the vascular dysfunction. In western diet-fed mice, ERα deletion prevented or reduced the beneficial effects of GSE on plasma triglycerides and visceral adiposity. ERα deletion also prevented/reduced the anti-oxidant effect of GSE in the liver, but did not affect its capacity to reduce oxidative stress in the heart and adipose tissue. In conclusion, dietary supplementation of GSE attenuated features of metabolic syndrome partially through ERα-dependent mechanisms. This report highlights the therapeutic potential of polyphenols, and especially extract enriched in procyanidin dimers, against the metabolic disorders associated with excessive energy intake.

## Introduction

Obesity represents a major health challenge in developed countries (Flegal et al., 2010; Malik et al., 2013) and contributes to increasing rates of cardiovascular and cerebrovascular events, type 2 diabetes, dyslipidaemia and metabolic diseases. Lifestyle modification remains the primary point of intervention for obesity management. However, pharmacological and dietary interventions represent promising strategies for both weight reduction and improving associated cardiometabolic risk (Ahluwalia et al., 2013; Sweeting et al., 2015). Among these dietary approaches, the use of nutraceuticals, has attracted great interest in the recent years (Magrone et al., 2013; Remely et al., 2015); including the supplementation with polyphenols, bioactive compounds from vegetables and fruits best known for their antioxidant properties (Duthie et al., 2003; Fraga et al., 2010). Different *in vitro* and *in vivo* studies have demonstrated a broad range of protective effects of polyphenols in the context of metabolic and cardiovascular diseases (Andriantsitohaina et al., 2012). We previously demonstrated in Zucker fatty rats that the red wine polyphenol (RWP) extract Provinols^TM^ improves glucose and lipid metabolism, as well as endothelial and cardiac functions without affecting the body weight gain (Agouni et al., 2009). Provinols^TM^ is mainly composed of the flavonoids flavanols and anthocyanins, which are described as the most effective classes of polyphenols (Chalopin et al., 2010).

Flavanols (cathechins and procyanidins) have antioxidant/free radical scavenging abilities (Galleano et al., 2010) and anti-inflammatory effects (Terra et al., 2011). They correct lipid abnormalities associated with obesity (Quesada et al., 2009; Caimari et al., 2013), prevent insulin resistance (Al-Awwadi et al., 2005; Montagut et al., 2010) and exert protective effects against cardiovascular diseases (Rasmussen et al., 2005; Corder et al., 2006). Anthocyanins limit weight gain, and improve lipid profiles, hepatic function (Wu et al., 2013) and insulin resistance (Nizamutdinova et al., 2009) associated with obesity. They also have antioxidant (Chiang et al., 2006; Azuma et al., 2008) and anti-inflammatory properties and trigger nitric oxide (NO^⋅^)-dependent vasorelaxation (Andriambeloson et al., 1998).

We have previously identified the molecular mechanisms involved in the regulation of vascular reactivity by RWP and anthocyanins. Provinols^TM^ and the antocyanin delphinidin modulate vascular relaxation mainly through the ERα. ERα is required for the activation of molecular pathways (including Src, ERK1/2, eNOS, and caveolin-1 phosphorylations) leading to the increase of endothelial NO^⋅^ production and endothelium-dependent vascular relaxation (Chalopin et al., 2010). ERα seems to be a key target for the beneficial effect of red wine polypenols. The critical role of ERα in the maintenance of metabolic homeostasis, the modulation of energy expenditure (Xu et al., 2015), the control of adipose tissue distribution (Heine et al., 2000), the regulation of insulin sensitivity, and protection against tissue inflammation (Ribas et al., 2010) is well documented.

We therefore hypothesized that ERα is one of the molecular targets triggering the beneficial effects of dietary supplementation of polyphenols extract against obesity-related cardiovascular and metabolic disorders. We tested this hypothesis in diet-induced obese mice supplemented with a grape seed extract (GSE) enriched in procyanidin dimers, flavan-3-ols described to be the most effective RWP on the endothelium (Aldini et al., 2003; Corder et al., 2006). Ovariectomized WT and ERα KO mice were fed with standard or western diet supplemented or not with GSE. The body weight, fat accumulation, glucose and lipid metabolism, cardiac and vascular functions, tissue NO^⋅^, and reactive oxygen species (ROS) productions and hepatic mitochondrial function were assessed.

## Materials and Methods

### Products

Grape seed extract (exGrape^®^SEED) was obtained from Grap’Sud (Cruviers-Lascours, France). The phenolic composition of dry powder in mg/g was: 50 catechins, 33.1 epicatechins, 1.7 epigallocatechin, 1.9 epigallocatechin 3-O-gallate, 16.8 B1 procyanidin dimers, 57.5 B2 procyanidin dimers, 7.7 B3 procyanidin dimers, 7.2 B4 procyanidin dimers, 3.7 B2 3’-O-gallate, 2.2 unknown dimer, 14.1 unknown trimer, 468.6 procyanidins polymers.

Grape seed extract was included by SAFE (Augy, France) into a chow supplied in powder form at the concentration of 150 mg/kg. Standard diet (U8960P version 0066) and western diet (U8958P version 0052) as well as those containing GSE were obtained from SAFE (Supplementary Table 1). Standard diet contains 5% of fat based on its composition in g/kg which represents 12% calories of total diet. With regard to western diet, it contains 21.5% of fat in g/kg that corresponds to 36.8% calories.

The concentration of extract used in present study was comparable with that described in our previous studies (Diebolt et al., 2001; Baron-Menguy et al., 2007; Agouni et al., 2009; Chalopin et al., 2010, 2014; Leonetti et al., 2017) and consequently it was comparable to the concentration that induced maximal relaxation of mouse aortic rings *ex vivo*. Also, it was consistent with a human consumption of one to two glasses of red wine per day and was in the range of that able to provide healthy effects on oxidative damage in humans (Covas et al., 2010).

### Ethics Statement

The animal protocol followed in the present study was approved by the local ethics committee (“Comité d’éthique en expérimentation animale Pays de la Loire”; CEEA.2011.40) in agreement with the guidelines and authorization with French Ministry of Agriculture regulations based on European Community.

### Animals and Protocol Design

Both ERα WT or KO mice (females of 8-week-old) were obteined from the Jackson Laboratory (Bar Harbor, ME, United States) and maintained at 23 ± 2°C under a 12 h light-dark cycle. Upon 1 week of acclimatization, mice were ovariectomized to avoid the cofounding effect of circulating estrogens in the effect of polyphenols. Seven days later, both strains were randomized divided into four groups standard diet, western diet (data concerning these mice have previously published by Leonetti et al. (2017), or standard diet and western diet containing GSE at the concentration of 150 mg/kg during 12 weeks (WT standard diet *n* = 5, WT standard diet+GSE *n* = 5, WT western diet *n* = 5, WT western diet+GSE *n* = 5, KO standard diet *n* = 5, KO standard diet+GSE *n* = 5, KO western diet *n* = 4, KO western diet+GSE *n* = 5). Mice were allowed ad libitum access to water and diets.

Mouse weight was measured weekly. One week before sacrifice, mice underwent an echocardiography. After treatment, mice were maintained in fasting conditions overnight (∼12 h) before sacrifice, then they were euthanized and the visceral and subcutaneous fat mass, liver and heart were removed from animals and weighted. The adiposity was calculated as the total adipose tissue weight (the sum of the visceral and subcutaneous adipose tissues) vs. total body weight. Fragments of tissue of each animal were frozen at -80°C for further analysis.

### Biochemical Analyses

Blood was collected by cardiac puncture at sacrifice (upon a fasting condition of ∼12 h). It was centrifuged for 15 min at 950 *g* and room temperature to obtain plasma. The evaluation of fasting glucose, triglycerides, total cholesterol, low density lipoprotein (LDL)-cholesterol, and high density lipoprotein (HDL)-cholesterol was performed using Konelab^TM^ 20 Clinical Chemistry Analyzer (Thermo Fisher Scientific^TM^, Waltham, MA, United States).

### Echocardiography

Transthoracic echocardiography was performed in mice anesthetized with isoflurane using a VEVO 770 ultrasound echograph from FUJIFILM Visualsonics (Toronto, ON, Canada) with a 30-MHz imaging transducer. Briefly, a two-dimensional short axis view of the left ventricle was obtained in order to record M-mode tracings. LVEDD, LVESD, and COI were evaluated (Agouni et al., 2009; Leonetti et al., 2017).

### *Ex Vivo* Vascular Reactivity

After dissection, the aortic rings were mounted on a wire myograph (Danish Myo Technology, Aarhus, Denmark) and filled with Krebs solution with the following composition in mmol/L: NaCl 130, NaHCO_3_ 14.9, KCl 3,7, KH_2_PO_4_ 1.2, MgSO_4_.7H_2_O 1.2, CaCl_2_.H_2_O 1.6, glucose 11 (37°C, 95% O_2_-5% CO_2_) (Agouni et al., 2007). The maximal contractile capacity of the vessel was tested by challenging the artery with the combination of depolarizing solution, [i.e., KCl (100 mmol/L)-Krebs solution], and a maximally active concentration of the thromboxane analog U46619 (Sigma-Aldrich, Saint-Quentin-Fallavier, France). The presence of functional endothelium was assessed in all preparations by the ability of Ach (Sigma-Aldrich) to induce more than 60% relaxation of vessels pre-contracted to 80% of their maximal response with U46619.

Then, the concentration-response curves to Ach were constructed by cumulative addition of the agonist (1 nmol/L–10 μmol/L) in aortas with functional endothelium pre-contracted with U46619 (0.1 μmol/L). The vascular reactivity was assessed by cumulative addition of serotonin (5-HT, 10 nmol/L–30 μmol/L; Sigma-Aldrich) in vessels with functional endothelium.

### Evaluation of Nitric Oxide (NO^⋅^) and Reactive Oxygen Species (ROS)

Electron paramagnetic resonance evaluation of NO^⋅^ production in the aorta, visceral, and subcutaneous adipose tissues, heart, liver, and skeletal muscle was performed using DETC (Sigma-Aldrich) as a spin trap. ROS production was evaluated using deferoxamine chelated Krebs-Hepes solution containing CMH (500 μmol/L, Noxygen, Mainz, Germany), deferoxamine (25 μmol/L, Sigma-Aldrich), and DETC (5 μmol/L). Both measurements were carried out using a table-top x-band Miniscope MS200 Spectrometer (Magnettech, Berlin, Germany). Obtained signals were expressed in A.U. as data were corrected for total amplitude/mg weight of dried tissues as previously described (Agouni et al., 2009).

### Evaluation of Hepatic Mitochondrial Enzyme Activities

The activities of CS, complex I, II and IV on liver homogenates were performed spectrophotometrically at 37°C. Values were expressed in nmol of product formed per minute and per mg of protein in homogenates as previously described (Mingorance et al., 2012).

### Statistical Analysis

Data are expressed as mean ± SEM. and *n* represents the number of animals. The statistical software GraphPad Prism 6.0 (GraphPad Software Inc., San Diego, CA, United States) and R were used for data analysis. It was assessed that data follow a normal distribution by Shapiro–Wilk test. The difference between groups was performed by one-way ANOVA. If the ANOVA demonstrated a significant interaction between variables, *post hoc* analyses were performed by the multiple-comparison Sidak’s test.

When random effects are supposed in our analyses, we performed a linear mixed model. This kind of model allows us to take into account both variabilities (inter and intra observation). These analyses are performed using package lme4 from R statistical software. If coefficient associated with the variable of interest is significant, then we will realize *post hoc* analyses followed by Sidak correction. *P* < 0.05 was considered to be statistically significant.

## Results

### Effects of GSE Supplementation on Body Weight Gain and Organ Weights

We evaluated the body weight gain and organ weights after 2 weeks of western diet supplemented or not with GSE.

**FIGURE 1:**
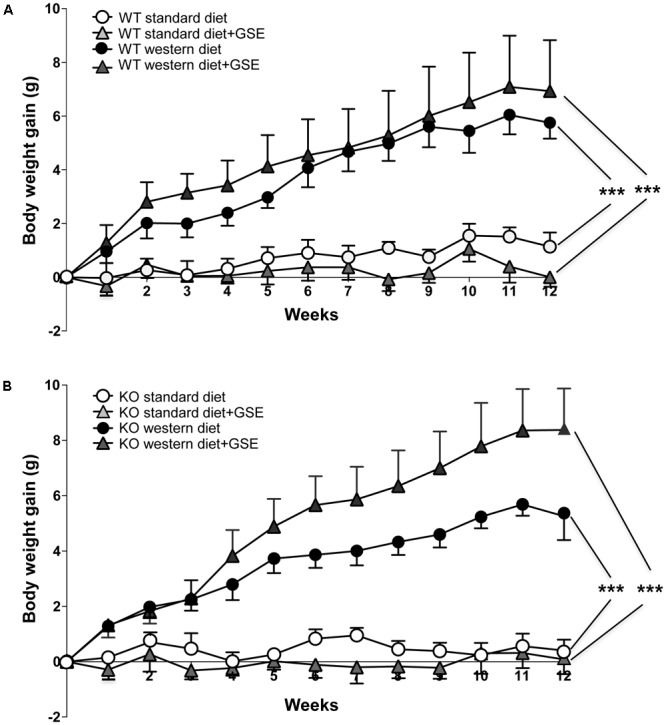
The evolution of body weight gain **(A,B)** of estrogen receptor alpha (ERα) wild type (WT) **(A)** and knockout (KO) **(B)** mice receiving standard diet, western diet, or standard diet and western diet containing grap seed extract (GSE) at the concentration of 150 mg/kg during 12 weeks. The body weight was recorded twice a week. The data are expressed as the mean ± SEM. Statistical analyses were performed by a linear mixed model and *post hoc* analyses followed by Sidak correction, ^∗∗∗^*P* < 0.001.

In ERα WT mice, western diet induced a time-dependent increase in body weight compared to standard diet-fed mice. The final body weight was increased in western diet-fed mice compared to standard diet-fed mice. This was associated with higher adiposity (**Table 1**). Interestingly, GSE supplementation partially prevented the fat accumulation induced by western diet (**Table 1**) without affecting the body weight gain (**Figure 1A**).

**Table 1:** Animal characteristics following 12 weeks of diets.

	WT				KO			
	Standard diet	Standard diet+GSE	Western diet	Western diet+GSE	Standard diet	Standard diet+GSE	Western diet	Western diet+GSE
Final bw (g)	20.09 ± 0.56	20.2 ± 0.38	26.42 ± 0.56^∗^	26.94 ± 2.25††	20.54 ± 0.93	20.28 ± 0.62	23.7 ± 1.37	29.62 ± 1.99£££$
Liver weight (% of bw)	6.04 ± 0.32	5.86 ± 0.15	6.30 ± 0.30	6.18 ± 0.51	4.42 ± 0.19^∗∗^	4.51 ± 0.21†	4.54 ± 0.21##	4.33 ± 0.22¥¥¥
Heart weight (% of bw)	0.64 ± 0.02	0.63 ± 0.01	0.53 ± 0.03	0.53 ± 0.04	0.46 ± 0.02^∗∗^	0.52 ± 0.04	0.47 ± 0.04	0.40 ± 0.01
Adiposity (% of bw)	5.27 ± 0.2	4.50 ± 0.14	12.36 ± 1.35^∗∗∗^	8.50 ± 0.8†#	4.71 ± 0.5	4.28 ± 0.34	10.6 ± 1.45§§§	12.65 ± 0.9£££¥

*The table shows the weight of mice, heart, visceral and subcutaneous adipose tissues, and liver of estrogen receptor alpha (ERα) WT or KO mice fed with standard diet, western diet, standard diet + GSE, or western diet + GSE for 12 weeks. The data are given as the mean ± SEM. GSE, grap seed extract; WT, Wild Type: KO, Knockout; bw, body weight. Statistical analyses are performed by one-way ANOVA and Sidak’s post hoc test, ^∗^P < *0,05, ^∗∗^P* < *0.01, ^∗∗∗^P* < *0.001 vs. WT standard diet group; †P* < *0,05, ††P* < *0.01 vs. WT standard diet+GSE group; #P* < *0.05, ##P* < *0.01 vs. WT western diet group; §§§P* < *0.001 vs. KO standard diet group; £££P* < *0.001 vs. KO standard diet+GSE; $P* < *0,05 vs. KO western diet; ¥ P* < *0,05, ¥¥¥P* < 0.001 vs. WT western diet+GSE*.

Likewise, the ERα KO mice group fed with western diet had an increase of body weight compared to standard diet-fed mice (**Figure 1B**). This was also associated with an increased adiposity index (**Table 1**). GSE did change neither the body weight nor the fat accumulation of KO mice fed with western diet (**Figure 1B**). ERα deletion enhanced the adiposity index of mice fed with GSE (**Table 1**).

The liver and heart weights were not significantly modified independently of diets in the two strains. However, ERα deletion decreased liver and heart weight in mice fed with standard diet; as well as the liver weight of standard diet-fed mice supplemented with GSE (**Table 1**).

These data suggest that, although the GSE did not affect body weight gain, it was able to partially prevent fat accumulation in western diet-fed mice through a mechanism that requires ERα.

### Effects of GSE Supplementation on Glycaemia and Lipid Profile

**FIGURE 2:**
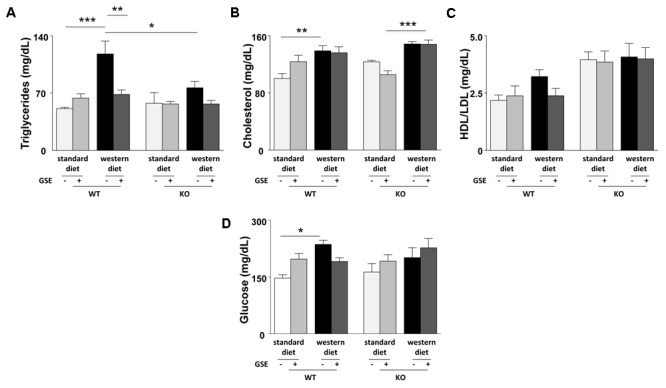
Circulating levels of triglycerides **(A)**, total cholesterol **(B)**, ratio between LDL- and HDL-cholesterol **(C)**, and glucose **(D)** were evaluated in fasting plasma of ERα WT and KO mice receiving standard diet, western diet, or standard diet and western diet containing GSE at the concentration of 150 mg/kg during 12 weeks. Blood was collected by cardiac puncture at sacrifice. The data are expressed as the mean ± SEM. Statistical analyses were performed by one-way ANOVA and and *post hoc* analyses followed by Sidak correction, ^∗^*P* < 0.05, ^∗∗^*P* < 0.01, ^∗∗∗^*P* < 0.001.

In ERα WT mice, western diet increased significantly the plasma levels of triglycerides, cholesterol and glucose without affecting the HDL/LDL ratio (**Figures 2A–D**). GSE did not modify circulating parameters in mice fed with standard diet but significantly reduced the plasma level of triglycerides in western diet-fed mice without significantly altering those of cholesterol, HDL/LDL and glucose (**Figures 2A–D**).

Conversely, no differences were observed between the lipid and glucose profiles of ERα KO mice fed with western diet compared to standard diet. However, ERα deletion decreased trygliceride levels of western diet-fed mice compared to western diet-fed WT mice. The GSE supplementation had no effect on the circulating lipid and glucose profiles of ERα KO mice fed with either standard diet orwestern diet.

Consequently, in the present experimental model, GSE did not affect glucose metabolism but prevented hypertriglyceridemia by a mechanism dependent to ERα.

### Effects of GSE Supplementation on the Aorta

**FIGURE 3:**
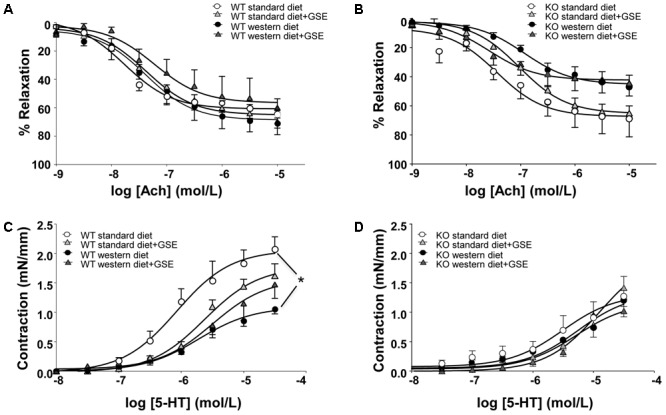
Concentration-response curves to Ach **(A,B)** and 5-HT **(C,D)** in aortic rings from ERα WT **(A,C)** and KO **(B,D)** mice receiving standard diet, western diet, or standard diet and western diet containing GSE at the concentration of 150 mg/kg during 12 weeks. The data are expressed as the mean ± SEM. Statistical analyses were performed by a linear mixed model and *post hoc* analyses followed by Sidak correction, ^∗^*P* < 0.05.

In ERα WT and KO mice, endothelium-dependent relaxation to Ach was neither affected by the diet nor the supplementation with GSE (**Figures 3A,B**). However, a significant reduction of Ach-induced relaxation was observed in western diet-fed KO mice when compared to western diet-fed WT mice (*p* < 0.0001).

In ERα WT mice fed with western diet, we observed a hyporeactivity to 5-HT compared to the standard diet group; without any effect of GSE supplementation in either groups (**Figure 3C**). Conversely, neither the diet nor the supplementation with GSE affected the contractile response to 5-HT in KO mice (**Figure 3D**). However, mice lacking ERα showed a reduced contractile response to 5-HT when compared to WT ERα mice fed with standard diet.

Taken together, the experimental model of obesity used in this study is associated with vascular function alterations: western diet induced vascular hyporeactivity in ERα WT mice and tended to impair endothelium-dependent relaxation in ERα KO mice. The treatment of GSE was not able to correct these vascular alterations.

### Effects of GSE Supplementation on the Heart

As indicated in **Table 2**, the heart structure and function was affected neither by the strain, the diet, nor the GSE supplementation.

**Table 2:** Cardiac function following 12 weeks of diets.

	WT				KO			
	Standard diet	Standard diet+GSE	Western diet	Western diet+GSE	Standard diet	Standard diet+GSE	Western diet	Western diet+GSE
LVEDD (mm)	3.5 ± 0.1	3.4 ± 0.1	3.7 ± 0.1	3.6 ± 0.2	3.4 ± 0.07	3.4 ± 0.2	3.2 ± 0.1	3.6 ± 0.1
LVESD (mm)	2.2 ± 0.1	2.1 ± 0.1	2.6 ± 0.1	2.5 ± 0.3	2.1 ± 0.03	2.2 ± 0.4	1.8 ± 0.06	2.0 ± 0.05
EF (%)	69.1 ± 2.8	68.8 ± 2.6	65.3 ± 4.81	66.6 ± 4.13	70.4 ± 1.0	69.7 ± 3.6	74.7 ± 2.3	74.1 ± 2.3
COI (mL/min/g)	0.8 ± 0.04	0.8 ± 0.04	0.7 ± 0.07	0.7 ± 0.04	0.8 ± 0.1	0.8 ± 0.1	0.8 ± 0.1	0.8 ± 0.2

*The table shows the LVEDD, LVESD, EF, COI in ERα WT or KO mice fed with standard diet, western diet, standard diet+GSE, or western diet+GSE for 12 weeks. The data were given as the mean ± SEM. Abbreviations: WT, Wild Type: KO, Knockout; GSE, grap seed extract; LVEDD, left ventricular end-diastolic dimension; LVESD, left ventricular end-systolic dimension; EF, ejection fraction; COI, cardiac output index*.

### Effects of GSE Supplementation on the Liver Enzymes

**FIGURE 4:**
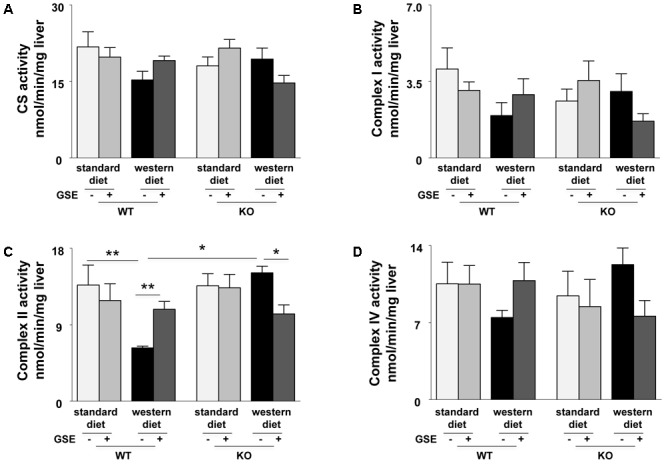
Measurement of enzymatic activities of mitochondrial cytrate synthase (CS) **(A)** and respiratory chain complexes (I, II, and IV) **(B–D)** in liver from ERα WT and KO mice receiving standard diet, western diet, or standard diet and western diet containing GSE at the concentration of 150 mg/kg for 12 weeks. The data are expressed as the mean ± SEM. Statistical analyses were performed by one-way ANOVA and *post hoc* analyses followed by Sidak correction ^∗^*P* < 0.05, ^∗∗^*P* < 0.01.

In ERα WT mice, western diet induced a significant reduction of complex II activity (**Figure 4C**). GSE supplementation tended to enhance the mitochondrial complexes activity of mice fed with western diet and significantly increased complex II activity. However, it did not have any effect in the mice fed with standard diet.

In ERα KO mice, the mitochondrial complexes activities were not affected by the diet. GSE had no effect in mice fed with standard diet; but tended to decrease the CS, complex I and complex IV activities and significantly reduced complex II activity in mice fed with western diet (**Figures 4A–D**).

These results suggest that GSE improved hepatic mitochondrial function by a mechanism dependent to ERα.

### Effect of GSE Supplementation on NO^⋅^ Production

**FIGURE 5:**
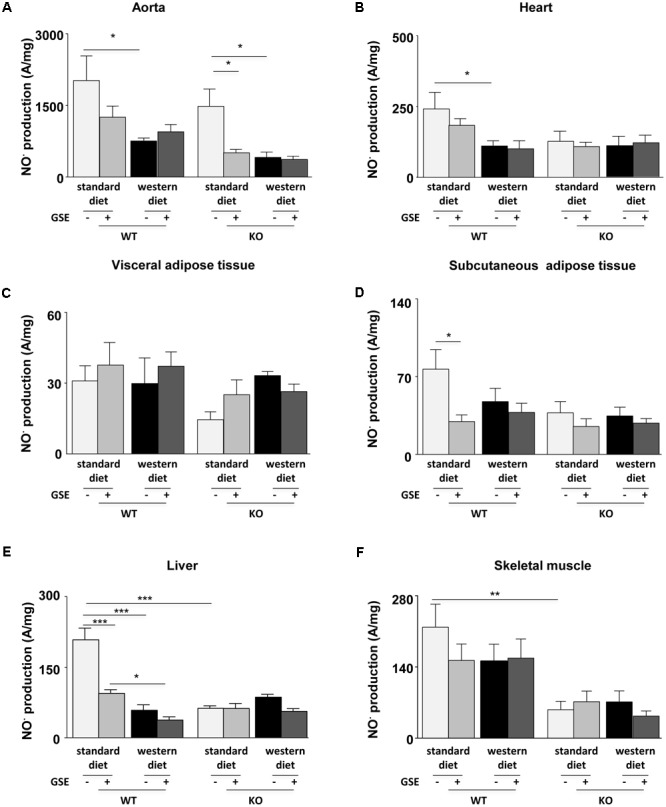
Measurement of *in situ* nitric oxide (NO^⋅^) production by electron paramagnetic resonance (EPR) in aorta **(A)**, heart **(B)**, visceral **(C)**, and subcutaneous **(D)** adipose tissues, liver **(E)**, and skeletal muscle **(F)** from ERα WT and KO mice receiving standard diet, western diet, or standard diet and western diet containing GSE at the concentration of 150 mg/kg during 12 weeks. The data were expressed as the mean ± SEM. Statistical analyses were performed by one-way ANOVA and *post hoc* analyses followed by Sidak correction, ^∗^*P* < 0.05, ^∗∗^*P* < 0.01, ^∗∗∗^*P* < 0.001.

In ERα WT and KO mice, western diet reduced NO^⋅^ production in the aortas; that was not prevented by GSE supplementation (**Figure 5A**). However, GSE supplementation significantly reduced NO^⋅^ production in the aorta of KO mice fed with standard diet.

In the heart, neither the strains nor the GSE supplementation affected NO^⋅^ production. However, we observed a significant reduction of NO^⋅^ production in the heart of WT mice fed with western diet compared to standard diet (**Figure 5B**).

The NO^⋅^ production in the visceral adipose tissue was not affected by any of the conditions (**Figure 5C**). In the subcutaneous adipose tissue, neither the strains nor the diet affected NO^⋅^ production. However, we observed that GSE supplementation significantly reduced NO^⋅^ production in the subcutaneous adipose tissue of WT mice fed with standard diet (**Figure 5D**).

In the liver of ERα WT mice, western diet significantly decreased NO^⋅^ production when compared to standard diet-fed mice. This was not affected by GSE supplementation. However, GSE supplementation significantly reduced NO^⋅^ production in the liver of WT mice fed with standard diet. In ERα KO mice, neither the diet nor the GSE supplementation affected NO^⋅^ production. However, hepatic NO^⋅^ level of mice lacking ERα and fed with standard diet was markedly reduced compared to the corresponding WT group (**Figure 5E**).

Finally, in the skeletal muscle, neither the diet nor the GSE supplemetation affected NO^⋅^ production. However, we observed a significant reduction of NO^⋅^ production KO mice fed with standard diet compared to WT (**Figure 5F**).

### Effect of GSE Supplementation on ROS Production

**FIGURE 6:**
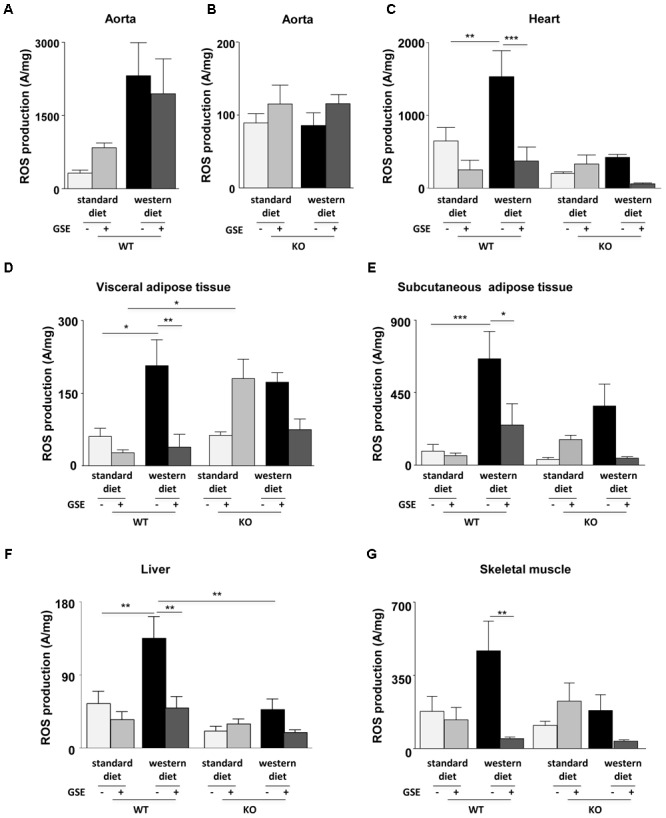
Measurement of *in situ* ROS production by EPR in aorta **(A,B)**, heart **(C)**, visceral **(D)**, and subcutaneous **(E)** adipose tissues, liver **(F)**, and skeletal muscle **(G)** from ERα WT and KO mice receiving standard diet, western diet, or standard diet and western diet containing GSE at the concentration of 150 mg/kg during 12 weeks. The data were expressed as the mean ± SEM. Statistical analyses were performed by one-way ANOVA and *post hoc* analyses followed by Sidak correction, ^∗^*P* < 0.05, ^∗∗^*P* < 0.01, ^∗∗∗^*P* < 0.001.

Reactive oxygen species production in aorta was not significantly modified independently of diets in the two strains (**Figures 6A,B**).

Western diet increased ROS production in the heart of ERα WT mice. GSE supplementation did not affect the ROS level in standard diet mice, but significantly reduced ROS production in the heart from western diet mice (**Figure 6C**). In ERα KO mice, western diet did not significantly modify ROS production. GSE supplementation did not affect ROS production in mice fed with standard diet wheras it trended to decrease ROS level in western diet-fed KO group.

In ERα WT mice, western diet significantly increased ROS production in the visceral adipose tissue. GSE supplementation did not modify ROS production in WT standard diet mice, in contrast it significatly reduced the level of ROS in western diet fed-mice (**Figure 6D**). In ERα KO mice, western diet did not significantly modified ROS production; GSE supplementation tended to enhance oxidative stress in mice fed with standard diet and failed to prevent the increase of ROS induced by western diet. KO standard diet+GSE group showed an increase of ROS production when compared to corresponded WT group.

As already observed in the visceral adipose tissue, western diet increased ROS production in the subcutaneous adipose tissue (**Figure 6E**) from ERα WT mice. GSE supplementation did not affect the ROS production in standard diet mice but reduced significantly the ROS level in mice receiving western diet. In ERα KO mice, western diet did not affect ROS production; GSE supplementation did not modify the ROS level of mice receiving standard diet diet and tended to reduce ROS level of mice receiving western diet.

Western diet significantly increased ROS production in the liver from ERα WT mice. GSE supplementation did not modify ROS level in standard diet mice; instead the supplementation of GSE to western diet prevented oxidative stress. In ERα KO mice, ROS production was not significant modified independently of diets. However, we observed a reduction of oxidative stress in ERα KO in mice fed with western diet compared to corresponded WT group (**Figure 6F**).

Finally, in the skeletal muscle of ERα WT mice, ROS production was increased in obese mice compared with mice fed with standard diet but not statistically significant. GSE supplementation did not modify the ROS level in standard diet mice; in contrast it significantly reduced that of mice fed with western diet. In ERα KO mice, western diet did not significant affect the ROS production. GSE supplementation did not modify oxidative stress in mice fed with standard diet, however, it tended to decrease ROS level in western diet group (**Figure 6G**). These results suggest that, in the heart, GSE was able to normalize the increased level of ROS induced by western diet through a mechanism independent of ERα. Also, GSE supplementation prevented oxidative stress induced by western diet in the visceral and subcutaneous adipose tissues as well as in the muscle by a mechanism independent to ERα. At contrary, in the liver, GSE supplementation protected against western diet-induced oxidative stress by a mechanism dependent to ERα.

## Discussion

In the present study, we provide evidence that the supple mentation of GSE significantly improved obesity-associated vascular and metabolic disorders. Although the GSE did not affect the increased body weight induced by western diet, it was able to partially prevent the fat accumulation and displayed antioxidant protection in the subcutaneous and visceral adipose tissues. Furthermore, GSE treatment improved plasma lipid profiles by preventing the western diet-induced hypertriglyceridemia, without affecting the cholesterol level and HDL/LDL ratio. Additionally, GSE decreased ROS production in the heart, liver, and skeletal muscle. In the liver, this effect was associated with an improvement of hepatic enzyme activities.

In accordance with previous report (Leonetti et al., 2017), the diet-induced obesity model used in our study increased mice’s body weight and adiposity, induced hypertriglyceridemia, hypercholesterolemia and hyperglycemia, and elevated oxydative stress in several organs and tissues (heart, visceral and subcutaneous adipose tissues, liver, and skeletal muscle). However, the western diet did not affect cardiac and endothelial functions but induced vascular hypo-reactivity in response to 5-HT.

The main objective of this study was to investigate the effects of oral administration of GSE enriched the flavan-3-ols procyanidin dimers in an experimental model of obesity. Here, we showed that GSE was not able to decrease the weight gain in response to the western diet, in line with previous studies using RWP in mice fed with western diet (Leonetti et al., 2017) or in Zucker fatty rats (Agouni et al., 2009).

Although GSE supplementation did not affect the body weight gain, it partially reduced fat accumulation induced by western diet. Adipose tissue plays a critical role in the regulation of energy balance. Its primary metabolic role is to store nutrients in the form of triglycerides and to mobilize fatty acids according to metabolic needs. In the present study, we found that the inhibition of fat deposition by GSE was associated with a reduction of plasma triglyceride levels. GSE did not affect the level of cholesterol and HDL/LDL ratio. Similar results were observed in mice fed with western diet and supplemented with a RWP (Leonetti et al., 2017). Since both extracts are rich in flavanols, these observations highlight a potential role of this class of polyphenols in correcting hypertriglyceridemia, an independent risk factor for the development of cardiovascular diseases.

In accordance with these results, it has been previously reported that the use of grape seed procyanidin extract improves plasma lipid profile and reduces plasma triglyceride levels (Pinent et al., 2004; Al-Awwadi et al., 2005; Quesada et al., 2009). In particular, the *in vivo* hypotriglyceridemic effect of dietary procyanidins involves the activation of the farnesoid X receptor, the subsequent upregulation of the nuclear receptor small heterodimer partner expression and the downregulation of SREBP1 expression (Del Bas et al., 2009). Another study also reports that the reduction of the lipid content in white adipose tissue and the improvement of plasma lipid profile induced by grape seed procyanidin traitement involve the activation of both β-oxidation and the glycerolipid/free fatty acid cycle (Caimari et al., 2013).

Beside its effect on lipid content, GSE also reduced the oxidative stress induced by western diet in both visceral and subcutaneous adipose tissues. It has been reported that the increased production of ROS in the obese white adipose tissue is associated with a decrease of the adipocytes’ antioxidant defenses (Le Lay et al., 2014). It has also been described that flavanol antioxidant property results from their ability to directly scavenge free radicals and/or chelate metals, or via indirectly modulating pro-oxidant enzymes (Fraga, 2007; Fraga and Oteiza, 2011). Finally, the treatment of adipocytes with flavanol-rich fruit extract has been shown to decrease ROS production and the gene expression of adipokine in adipocytes. These effects are associated with the downregulation of the transcriptional activity of NF-κB and the phosphorylation of ERK (Sakurai et al., 2008, 2010).

Grape seed extract also reduced oxidative stress without changing NO^⋅^ production in the heart, although it did not affect the cardiac structure and function. These results were similar to our previous study using RWP under the same experimental condition (Leonetti et al., 2017). It was also previously reported that the treatment of high-fructose fed rats with GSE reduces overproduction of ROS in cardiac tissue associated with a reduction of NADPH oxidase expression (Al-Awwadi et al., 2005). Consequently, the ability of GSE to improve oxidative stress in the heart may be linked either to the capacity of procyanidins to scavenge free radicals or to their ability to reduce the expression of pro-oxidant enzymes.

Interestingly, the antioxidant effects of GSE in the liver were associated with an improvement of complexe II activity in WT western diet-fed mice. In the liver, the obese state promotes lipogenesis and elicits mitochondrial dysfunction resulting in hepatic fatty acid and lipid overload. In the long term, these alterations in lipid homeostasis can promote oxidative stress and lipid peroxidation, leading to inflammation and fibrosis (Diebolt et al., 2001; Sanyal et al., 2001). Obesity may interfere with mitochondrial bioenergetics by affecting cellular respiratory functions and oxidative pathways. In the present study, we provided evidence that supplementation with GSE increased mitochondrial complex II activity in the liver of WD-induced obese mice. This could in turn improve the mitochondrial respiratory capacity. In line with these findings, it has been previously observed that the intake of procyanidin-rich extracts improves mitochondrial function in the skeletal muscle and brown adipose tissue in an experimental model of obesity (Pajuelo et al., 2012; Casanova et al., 2014). It is also described that the activity of the respiratory chain complexes is closely associated with mitochondrial ROS production (Le Lay et al., 2014). Accordingly, we observed that GSE-induced improvement of mitochondrial function was associated with a reduced of WD-induced oxidative stress in the liver. The antioxidant effect of GSE in the liver may also be due to the ability of the extract to modulate the expression and activity of antioxidant enzyme systems, as previously reported (Puiggros et al., 2005; Kim et al., 2013). One can hypothesize that the increased activity of the mitochondrial complex chain could also increase the oxidation of lipids and therefore prevent the rise of hepatic disorders such as steatosis.

We also observed that GSE prevented oxidative stress in the skeletal muscle from WD mice. In accordance with our data, a recent study reported that the administration of grape polyphenols in a mouse model of chronic high-grade inflammation reduces muscle atrophy and prevents ROS damage through the reduction of oxidized mitochondrial proteins, an improvement of mitochondrial function and a significantly reduction of caspases-9 and 3 activation (Lambert et al., 2015). Moreover, the treatment of diet-induced obese rats with grape seed proanthocyanidins extract improves muscle function by activating β-oxidation and by increasing mitochondrial functionality (Casanova et al., 2014).

Despite strong antioxidant effects, GSE supplementation did alter neither the endothelial function, nor the vascular reactivity, independently of the diet used. In accordance with these data, we recently reported that RWP do not alter responses to Ach and 5-HT, although it enhances NO^⋅^ production and reduces ROS in aortas (Leonetti et al., 2017). It should be noted that GSE neither affected NO^⋅^ nor ROS production in the aorta in the present study.

Finally, the main finding that has emerged from the present study was the identification of ERα as an important target involved in the reduction of fat accumulation and inprovement of hepatic function induced by GSE. Indeed, ERα deletion prevented the GSE-induced decrease of fat accumulation, the correction of hypertriglyceridemia and liver oxidative stress as well as the restoration of hepatic mitochondrial function in response to western diet; highlighting the requirement of this receptor in these processes.

Similarly, we recently provided evidence that ERα is involved in the protective *in vivo* effects of RWP against western diet-induced metabolic disorders, especially against the liver nitrative stress and visceral adiposity (Leonetti et al., 2017). ERα is also a key regulator of mitochondrial respiration and is involved in the modulation of expression of antioxidant genes (O’Lone et al., 2007; Chen et al., 2009; Gupte et al., 2015). The ability of polyphenols to modulate the mitochondrial capacity through the activation of ERα has already been reported in previous studies (Lu et al., 2012; Duluc et al., 2013). Taken together, the present study provides additional informations about the role of ERα as a target for other class of polyphenols such as flavanols.

However, we found that the antioxidant effects of GSE in the visceral and subcutaneous adipose tissues, the heart and skeletal muscle are independent of ERα. One can hypothesize the involvement of the estrogen receptor β or GPR30, which are gene regulators, expressed in different target tissues of obesity (Barros and Gustafsson, 2011; Sharma and Prossnitz, 2016).

## Conclusion

**FIGURE 7:**
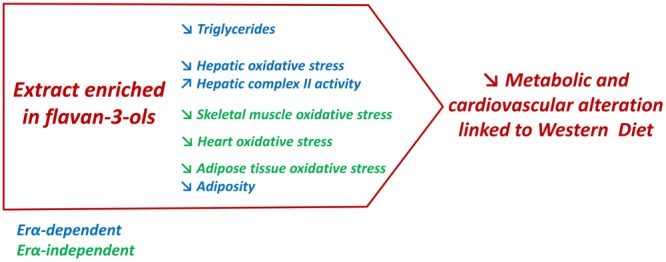
In western diet-fed mice, dietary supplementation with GSE reduced circulating triglycerides, liver oxidative stress and adiposity, and increased hepatic complex II activity via an ERα-dependent mechanism. However, in skeletal muscle, heart and adipose tissue, GSE supplementation decreased ROS production independently to ERα. Hence, the effects triggered by GSE attenuate most features of metabolic dysfunctions partially via ERα.

The present report highlighted the ability of a GSE enriched with the flavan-3-ols procyanidin dimers to attenuate obesity-associated disorders (**Figure 7**). The most important information provided in this study was the identification of ERα as an important target of GSE to reduce adiposity and improve liver functions in an experimental model of obesity. Additional studies are required to understand the molecular mechanisms involved in these beneficial effects. This study is a good starting point to promote the therapeutic potential of GSE against the cardiovascular and metabolic disorders associated with obesity.

## Author Contributions

RA: conceived the experiments. RA and MM: designed the experiments. DL, RS, NC, LV, CJ, and LD: performed the experiments. DL, RS, NC, CJ, and LD: acquired data. DL, RS, and NC: analyzed the data. CD: managed financial and administrative tasks. RS, MM, and RA: interpreted and discussed the results. DL, RS, MM, and RA: wrote and revised the manuscript. All authors read and approved the final manuscript.

## Conflict of Interest Statement

The authors declare that the research was conducted in the absence of any commercial or financial relationships that could be construed as a potential conflict of interest.

## Abbreviations

5-HT: serotonin
A: amplitude
A.U.: arbitrary units
Ach: acetylcholine
ANOVA: analysis of variance
bw: body weight
CMH: 1-hydroxy-3-methoxycarbonyl-2,2,5,5-tetramethylpyrrolidine
COI: cardiac output index
CS: citrate synthase
DETC: Fe^2+^-diethyldithiocarbamate
EF: ejection fraction
EPR: electron paramagnetic resonance
ERα: estrogen receptor alpha
GSE: grape seed extract
HDL: high density lipoproteins
KO: Knockout
LDL: low density lipoproteins
LVEDD: left ventricular end-diastolic dimension
LVESD: left ventricular end-systolic dimension
NO^⋅^: nitric oxide
ROS: reactive oxygen species
RWP: red wine polyphenols
WT: Wild Type
