# Supplementary material for: Extract Enriched in Flavan-3-ols and Mainly Procyanidin Dimers Improves Metabolic Alterations in a Mouse Model of Obesity-Related Disorders Partially via Estrogen Receptor Alpha
Source: Front Pharmacol. 2018 Apr 24;9:406. doi: 10.3389/fphar.2018.00406 (PMC5928481; doi:10.3389/fphar.2018.00406)
Supplement: Supplementary file 1 [file Table_1.DOCX]

**Supplemental Table 1**. Composition of diets

| Diet | standard  diet | standard diet+GSE | western  diet | western diet+GSE |
| --- | --- | --- | --- | --- |
| Composition, g/Kg |  |  |  |  |
| GSE | - | 0.15 | - | 0.15 |
| Sucrose | 207 | 207 | 340 | 340 |
| Dairy butter | 50 | 50 | 200 | 200 |
| Casein | 200 | 200 | 180.5 | 180.5 |
| Pregelatinized cornstarch | 400 | 400 | 145 | 145 |
| Pre-mixture of minerals | 70 | 70 | 70 | 70 |
| Crude cellulose | 60 | 59.85 | 50 | 49.85 |
| Pre-mixture of vitamins | 10 | 10 | 10 | 10 |
| DL-methionine | 3 | 3 | 3 | 3 |
| Cholesterol | - | - | 1.5 | 1.5 |
| Energy, Kcal/Kg |  |  |  |  |
| Protein | 735 | 735 | 664.4 | 664.4 |
| Fat | 423.3 | 423.3 | 1550.5 | 1550.5 |
| Carbohydrate | 2360.7 | 2360.7 | 2002.5 | 2002.5 |
| Energy, % |  |  |  |  |
| Protein | 21 | 21 | 16 | 16 |
| Fat | 12 | 12 | 37 | 37 |
| Carbohydrate | 67 | 67 | 47 | 47 |
